# Reporting of conflicts of interest and of sponsorship of guidelines in anaesthesiology. A cross-sectional study

**DOI:** 10.1371/journal.pone.0212327

**Published:** 2019-02-27

**Authors:** Damien Wyssa, Martin R. Tramèr, Nadia Elia

**Affiliations:** 1 Division of Anaesthesiology, Geneva University Hospitals, Geneva, Switzerland; 2 Faculty of Medicine, University of Geneva, Geneva, Switzerland; 3 Institute of Global Health, Faculty of Medicine, University of Geneva, Geneva, Switzerland; Local Health Authority of South Tyrol, ITALY

## Abstract

Guideline recommendations may be biased due to conflicts of interest (COI) of panel members and sponsorship of the guideline. Potential impact of COI, and their management, should be transparently reported. We analysed 110 guidelines published in ten anaesthesia journals from 2007 to June 2018. We report on the number (%) that 1) published COI disclosures; 2) in a distinct paragraph; 3) described and explained the COI of panel members, and 4) of the Chairperson; 5) reported and described the presence or absence and potential impact of a sponsor of the guideline on the recommendations; and 6) reported how COI were managed. COI were published in 70/110 (64%) guidelines; in a distinct paragraph in 25/70 (36%). Panel members reported having no COI in 27/70 (39%) guidelines, disclosed COI without describing their potential impact in 41/70 (59%), and described their potential impact in 2/70 (3%). Chairpersons were identified in 50 guidelines, 32 of which published COI disclosures; 16/32 (50%) reported having no COI, 14/32 (44%) disclosed COI without describing their potential impact, 1/32 (3%) described their impact and 1/32 (3%) made no statement regarding COI. Presence or absence of a sponsor of the guideline was reported in 40 guidelines; 12/40 (30%) declared none, 24/40 (60%) reported sponsoring without explanation of the potential impact, and 4/40 (10%) described the potential influence of the sponsor on the guideline recommendations. Seventy-five guidelines reported COI of panel members and/or sponsorship of the guideline but only seven described how the COI had been managed. Disclosures of COI of panel members and of sponsors of guidelines have increased over the 12 year period, but remain insufficiently described and their potential influence on the guidelines’ recommendations is poorly documented.

## Introduction

Guidelines aim to assist clinical decision-making based on high quality research evidence. It has been shown that conflicts of interest (COI) of panel members producing the guidelines exist [1][2], that they are often underreported [3][4] and that they may bias the guideline recommendations [5][6]. Besides, it has been suggested that financial relationships between organisations producing the guidelines and industry are common [7], and that they are also frequently underreported [4][6].

Recommendations regarding the management of COI state that panel members with COI should be excluded from the guideline process whenever possible [8][9] or, at least, that COI of panel members should be recorded and addressed [10]. Also, recommendations suggest that chairpersons should be free of any COI, and a neutral methodologist should be chosen as a co-chair when the chairperson has unavoidable direct or indirect COI [11]. Finally, it is recommended that guidelines should not be directly funded by industry [12], and the sponsor of a guideline should not influence the content of the guideline [10].

Although disclosures of COI are clearly a step towards increased transparency, and ought to be acknowledged, they often fail to clarify how the declared COI have, or could have, influenced the recommendations. For example, a panel member may report a relationship with a pharmaceutical company without declaring which specific product of this company is promoted by the guideline.

We set out to investigate how COI of panel members and chairpersons, as well as sponsorship of the guidelines, are reported in guidelines published in ten major anaesthesia journals over a twelve-year period.

### Objectives

Our primary objectives were to describe the number (%) of guidelines that

1. Published the panel members’ COI disclosures
2. Did so in a distinct, clearly identified, paragraph
3. Reported, and described the potential impact COI of panel members on the guideline recommendations
4. Reported, and described the potential impact of the COI of the chairperson on the guideline recommendations
5. Reported and described the potential impact of a sponsor of the guideline on the recommendations
6. Described how the COI, when present, have been managed.

Our secondary objective was to examine reporting trends over time.

## Methods

We followed the STROBE recommendations for the reporting of cross-sectional studies [13]. No ethical approval was required for this study.

### Study design

This study was designed as a cross-sectional analysis of systematically searched guidelines published from January 2007 to June 2018 in ten leading anaesthesia journals.

### Inclusion criteria

We used *InCites Journal Citation Reports* (Thompson Reuters) to identify the ten anaesthesia journals, excluding pain journals, with the highest impact factor (IF) in 2017. These were: *Anesthesiology* (IF 6.523), *British Journal of Anaesthesia* (6.499), *Anaesthesia* (IF 5.431), *Regional Anesthesia and Pain Medicine (*IF 4.382), *European Journal of Anaesthesiology* (IF 3.979), *Anesthesia & Analgesia* (IF 3.463), *International Journal of Obstetric Anesthesia* (IF 3.404), *Canadian Journal of Anesthesia* (IF 3.377), *Journal of Neurosurgical Anesthesiology* (IF 3.238) and *Minerva Anestesiologica* (IF 2.693). We then searched in Medline (via Pubmed) for all guidelines that had been published in these journals between 1^st^ of January 2007 and 30^th^ of June 2018 using two search strategies. In the first search strategy, we used the criterion [*name of the journal*] and the filters “Guideline” or “Practice guideline”. In the second, we used the criterion [*name of the journal*] AND (*guideline*[Title] OR *guidelines*[Title] OR *consensus statement*[Title]). We included reports with the term “guideline” or “consensus statement” in the title, or that were described as a “guideline” in the subtitle. We excluded guidelines on methodological (for instance, statistical) topics. When a guideline on a given topic was updated every year, published in the same format and in the same journal, we included the latest update and excluded previous versions. When a guideline on a given topic was published as multiple articles in the same issue of a journal, stratified according to the studied population for example, we examined all articles but counted them as one unique guideline.

### Setting

Searches were performed in July 2018 by one author (NE) and inclusion criteria were checked by a second author (DW). Data were extracted from July to September 2018 by one author (DW) and were checked by another (NE). Searches and access to guidelines were made through the library of the Medical Faculty of the University of Geneva, Geneva, Switzerland.

### Data extraction

We searched for specific keywords in the electronic document using the “Search for” tool in .pdf documents. For example, to search for statements regarding sponsorship of a guideline, we used the keywords “funding”, “financial”, “sponsor” or “support”. When none of the keywords was identified in the text, the complete guideline was read to make sure the information had not been missed.

### Variables

From each included guideline, we extracted information regarding the name of the journal, year of publication, number of panel members, name of the chairperson, and sources of sponsorship of the guideline. Sponsorship was categorised into four categories: academic or institutional, medical society, industry, or mixed (more than one source of funding disclosed). We specifically checked the six items described in Table 1. Additionally, we considered that a detailed description was needed on how COI and potential bias due to sponsorship of the guideline were managed if at least one panel member, or the chairperson of the guideline, declared having a COI, or if the guideline had received sponsorship. If the information was lacking for one or more of these items (for example, panel members declared lack of COI, but there was no statement regarding sponsorship of the guideline) we considered that the need for a detailed description of how COI were managed was “unclear”. If it was explicitly reported that none of the panellists had any COI and that no funding had been received, we considered that a description of how COI were managed was not needed.

**Table 1:** Degrees of quality of reporting of conflicts of interest.

	Worst case	Ambiguous	Adequate	Best case
**Reporting of disclosed COI**				
**1. Accessibility of disclosures**	Panel members' and chairperson's COI not mentioned	Panel members' and chairperson's COI available on request	Panel members' and chairperson's COI reported online	Panel members' and chairperson's COI reported in the published report
**2. Format of published COI disclosures**	Panel members' and chairperson's COI not reported	Panel members' and chairperson's COI reported but not in a separate paragraph	Panel members' and chairperson's COI reported in a separate paragraph, but not clearly identified	Panel members' and chairperson's COI reported in a separate paragraph clearly identified with the term "interest"
**Content of disclosure of COI **		** **	** **	** **
**3. Panel members**	Not disclosed	Disclosed without description of potential influence	Disclosed with description of potential influence	Disclosed that there were none (for all panel members)
**4. Chairperson**	Not disclosed	Disclosed without description of potential influence	Disclosed with description of potential influence	Disclosed that there were none
**Content of disclosure of sponsorship of the guideline**				
**5. Sponsor of guideline**	Not disclosed	Disclosed without description of potential influence	Disclosed with description of potential influence	Disclosed that there was none
**Description of how COI were managed**				
**6. Management of COI**	Not disclosed	Not disclosed and unclear if management required	Disclosed that COI were managed, without description	Disclosed that COI were managed, with description

COI = conflict of interest

### Bias

To limit bias in the selection of guidelines and data extraction, two authors separately performed the search strategies and the data extraction (DW, NE). Any discrepancy in data extraction was discussed with the third author (MRT) until a consensual decision could be reached.

### Study size

This investigation was designed as a descriptive study. There was no intention to search for associations. There was a pre-hoc assumption that the incidence of anaesthesia guidelines fulfilling all requirements of adequate reporting of COI would be low (<10%). In order to have results that may be generalisable to the entire field of anaesthesia, and to allow for examination of time trends, we decided to search in ten major anaesthesiology journals over a 12-year period.

### Statistical methods

Categorical variables are described as numbers (percentages) and a summary proportion of each variable over the whole period of time is provided. For the time trend analyses, variables are reported stratified according to three periods of publication (2007–2010; 2011–2014; 2015–2018). For this analysis, we dichotomised all six items described in Table 1 into two groups: Group 1 “best case” and “adequate” reporting; Group 2 “ambiguous” and “worst case” reporting. We computed the odds of “best-case or adequate” versus “ambiguous or worst case” reporting for each item, and we computed odds ratios (OR) with 95% confidence intervals (CI) comparing each time period with the baseline (2007–2010). We performed a similar analysis by using year of publication as a continuous variable. All analyses presented are two-sided, alpha level was fixed at 5% and analyses were performed using STATA 15 (StataCorp, 4905 Lakeway Dr, College Station, Tx 77845, USA).

## Results

### Selection of guidelines and descriptive data

**Fig 1:**
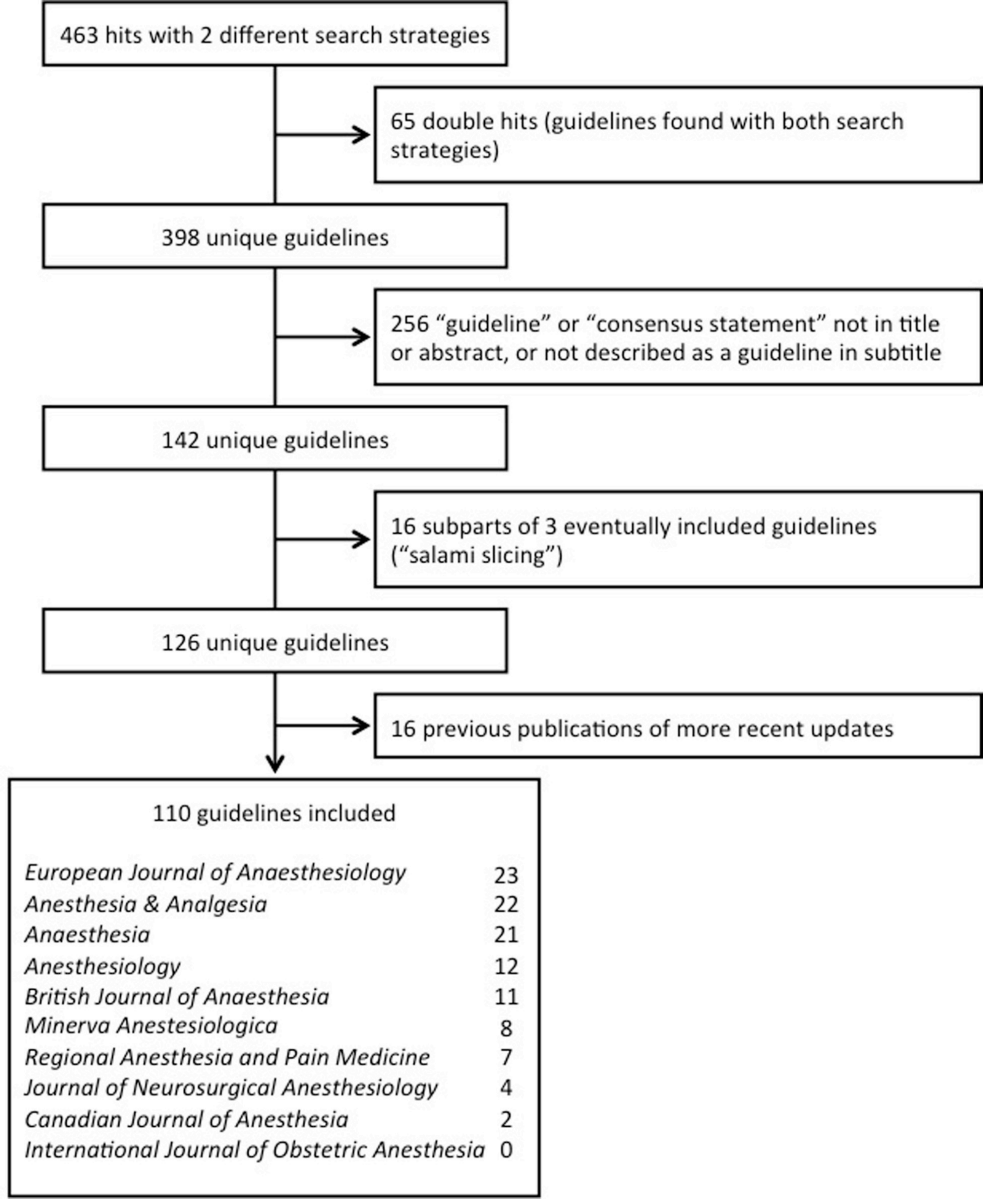
Flowchart or retrieved and eventually analysed guidelines.

Our two search strategies identified 463 references, of which we excluded 353 (Fig 1).

We eventually analysed 110 guidelines published from January 2007 to June 2018; 92% (101/110) were freely accessible. The number of guidelines published per year varied from 6 to 15 (median 9). The median number of panel members per guideline, including chairpersons, was 9 (interquartile range 7 to 13; min 1, max 34) (See S1 Table for a detailed description of all included guideline).

### Primary outcomes

#### 1. Accessibility of COI disclosures

**Fig 2:**
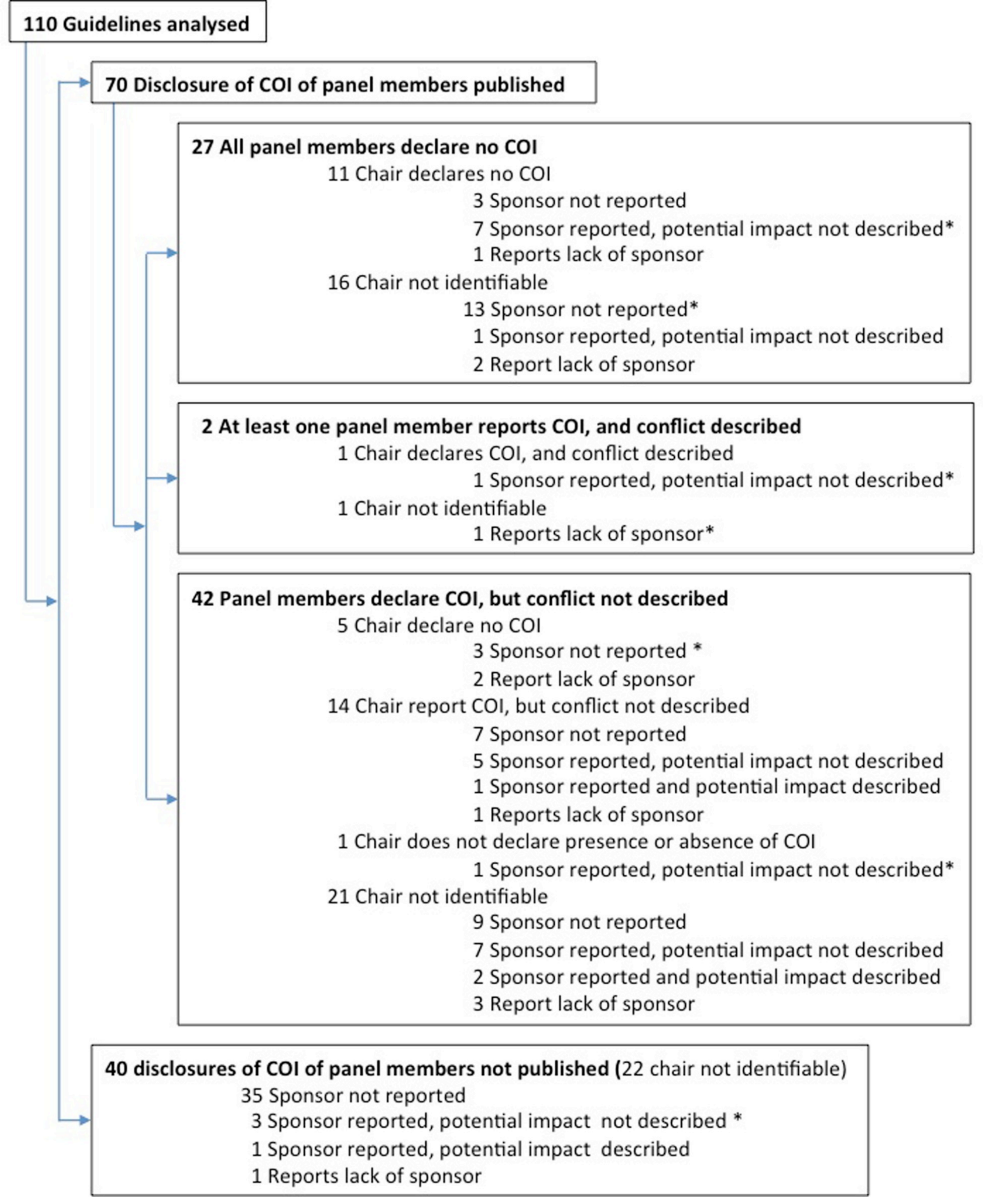
Disclosures of conflict of interest of panel members and chairs and sponsorship of guidelines. COI: conflict of interest; *Guidelines describing how COI were managed.

Seventy of 110 guidelines (64%) reported COI disclosures of panel members in the published guideline and 40 (36%) did not. Of the 40 guidelines that did not, one^E15^ published them on the journal’s website (freely accessible), one^E10^ stated that disclosures were available “on request”, and another ^E18^ published them on the journal’s website, but the internet link was not available (Fig 2).

#### 2. Format of published COI disclosures

Of the 70 guidelines that reported COI of panel members in the published report, 52 (74%) did so in a distinct paragraph. In 25 (36%), the paragraph was clearly identified and included the term “interest” (for example, “declaration of interest”, “conflict of interest”, or “competing interest”), in 27 (39%), the disclosures were reported under various paragraphs labelled such as “acknowledgement” or “appendix”, and in 18 (25%), the disclosures were not reported in a distinct paragraph, but were reported as a footnote on the title page.

#### 3. Content of disclosures of COI of panel members, when reported in the published guideline

Of the 70 guidelines which published COI of panel members, 27 (38%) reported that all panel members were free of any COI, two (3%) reported presence of COI of at least one panel member and described how the COI could have influenced the guideline’s conclusions, while 41 (59%) reported COI of panel members without further explanation (Fig 2).

#### 4. Content of disclosures of COI of chairpersons, when reported in the published guideline

A chairperson could be identified in 50 of the 110 guidelines. Among the 70 guidelines reporting on COI in the published report, a chairperson could not be identified in 38 (54%), the chairperson reported having no COI in 16 (23%), and reported having COI but provided no description of the potential impact of the COI on the guideline’s recommendations in 14 (20%). In one (1%), the chairperson reported COI and described their potential influence on the guideline recommendations. In another (1%), the chairperson did not report on presence or absence of COI (Fig 2).

#### 5. Content of disclosures of the sponsor of the guideline

Forty guidelines reported on presence or absence of a sponsor in the published guideline. Of these, 12 (30%) reported that there was no sponsor, 24 (60%) reported having received sponsorship but did not provide any explanation on how the sponsor may have influenced the recommendations, and four (14%) reported that the sponsorship was received but that it had no impact on the recommendations. Of the 28 guidelines reporting sponsorship, 17 (61%) declared sponsor from a medical society, five (18%) from academic or institutional funds, three (11%) from industry, and three (11%) from various sources (S2 Table).

#### 6. Management of COI of panel members and of sponsors of the guideline

In 35 of 110 (32%) guidelines, neither COI of panel members nor sponsors of the guidelines were disclosed. In the remaining 75 guidelines (68%), COI of panel members and/or a sponsor of the guideline were reported. Among these 75 guidelines, three (4%) explicitly reported that none of the panel members had any COI and that no sponsorship had been received for the development of the guideline. We considered that in these three guidelines there was no need to describe how COI had been managed. In 17 guidelines (23%), although panel members reported not having any COI, there was no statement regarding the presence or absence of sponsorship of the guideline (16), or the chairperson was not identified (1). For these guidelines, we considered that it was unclear whether or not a description of how COI had been managed was needed. For the remaining 55 guidelines, we deemed that a clear description of how COI had been managed was needed since COI of at least one panel member, of the chairperson, or the presence of a sponsor of the guideline was reported.

Seven guidelines described how COI had been managed (six of the 55 (11%) guidelines that we considered in need of a detailed description, and one that we considered “unclear”). The types of management described were to ask panel members with COI to abstain from voting, or to ask them to withdraw from discussions related to the topics in which he/she had a COI. In one guideline, an anonymous vote was held among the delegates of the medical society to decide the potential exclusion of panellists with COI. In another, the management consisted in submitting the entire guideline document to internal and external review and in one, the authors reported that “COIs were disclosed and managed” however without explaining how this was done (Fig 2 and S2 Table).

### Trend over time of reporting

The proportion of guidelines reporting disclosed COI and sponsors of the guideline in the published report increased over time (Tables 2 and S2). The proportion of guidelines reporting disclosures of COI of panel members in the published report has significantly increased from 28% in the period 2007–2010, to 87% in the period 2015–2018, p-trend <0.001. The proportion of guidelines reporting presence or absence of sponsorship of the guideline also increased significantly over time from 13% (2007–2010) to 54% (2015–2018), p-trend<0.001. There was no evidence that, among the guidelines that published COI disclosures, the format or content of the declaration had changed over time (S2 Table). When considering all the 110 guidelines however, the odds of having panel members (or chairpersons) disclosing no COI, or disclosing COI with a clear explanation of how the COI may have influenced the results (compared to those not reporting COI or reporting COI without explanation), have increased over time. Also, the odds of reporting on how the COI were managed improved over time, but remained marginal (Table 2).

**Table 2:** Trends of reporting of conflicts of interest and funding over time.

	n	Accessibility of disclosures		COI of panel members		COI of chairperson		Funding		Description of how COI were managed	
	total	n^1^/n^2^	OR (95%CI)	n^1^/n^2^	OR (95%CI)	n^1^/n^2^	OR (95%CI)	n^1^/n^2^	OR (95%CI)	n^1^/n^2^	OR (95%CI)
**2007–2010**	32	10/22	baseline	3/29	baseline	1/31	baseline	1/31	baseline	0/32	baseline
**2011–2014**	39	28/11	5.6 (2.0–15.6)	10/29	3.3 (0.83–13.4)	5/34	4.6 (0.5–41.2)	9/30	9.3 (1.1–78.0)	2/37	n/a
**2015–2018**	39	35/4	19.3 (5.4–69.0)	16/23	6.7 (1.74–25.9)	11/28	12.2 (1.5–100.4)	6/33	5.6 (0.64–49.5)	5/34	n/a
p^1^			<0.001		0.008		0.009		0.035		
**2007–2018**	110		1.52 (1.28–1.80)		1.26 (1.09–1.46)		1.36 (1.12–1.65)		1.18 (0.99–1.4)		4.15 (0.0–0.10)
p^2^			<0.001		0.002		0.002		0.058		0.018

n: number of guidelines analysed; COI: conflict of interest; n1: number of guidelines classified as "adequate" or "best case" according to Table 1; n2: number of guidelines classified as "ambiguous" or "worst case" according to Table 1; OR: odds ratio; CI: confidence interval; p1: p-value for trend of odds based on the three time periods; p2: p-value for trends of odds using year of publication as a continuous variable; n/a: OR not computable since baseline odds was 0.

## Discussion

### Main results

We set out to investigate how COI of panel members and sponsorship of guidelines have been reported in guidelines published in leading anaesthesia journals over the last 12 years. Although the proportion of guidelines that disclosed, in the published report, the COI of panel members has increased over time, less than half of them only did so in a distinct, clearly identifiable, paragraph. When COI were reported, it often remained unclear what the nature of the conflict was, and how it could have influenced the recommendations of the guidelines. Although half of the guidelines disclosed COI of panel members or the presence of a sponsor of the guideline, only seven described in the published report how the conflicts were managed. Finally, a minority of the panel members and chairpersons reported having no COI.

### Results in context

Although the impact of sponsors on the conclusions of clinical trials has been demonstrated [14][15], the impact of sponsors and of COI of panel members on the recommendations of guidelines has not yet been formally shown [6][16]. Recent scandals that were related to COI of panel members [17][18], some of which have led to the retraction of guidelines [19], suggest that this topic is sensitive, and highlights that transparent and explicit disclosures of COI of panellists are required. The introduction in the US of the “Sunshine Act” in 2010, which aims to openly disclose the relationships between pharmaceutical companies and physicians, also illustrates the importance given to the transparent disclosures of COI [20]. Recommendations on the management of COI during the guideline process have been proposed by the *Guideline International network* (GIN) and the *Committee on Conflict of Interest in Medical Research*, *Education*, *and Practice* of the US Institute of Medicine. The aim of such recommendations is to limit biases and to preserve scientific integrity. Both recommendations suggest that the chairpersons should be free of any COI. A chairperson’s COI may impact on the recommendations by the biased selection of panel members who are known to support a desired outcome [21]. Both also recommend that COI of all panel members should be declared, with a clear description of how each of the COI has been managed during the guideline process. Finally, presence or absence of a sponsor of the guideline should be clearly reported, and the potential impact of the sponsor on the guidelines must be explained [8][22]. Despite these recommendations, several studies have highlighted weaknesses in the management of COI in guidelines, and have called for improvement [1][7][11][23].

Reporting of COI in guidelines has been the object of previous studies. Some have focused on the proportion of guidelines reporting (or not reporting) COI of panel members. In 2001, a study of guidelines in internal medicine that were published between 1994 et 1999, reported that only 3.7% had reported COI of panel members [24]. Ten years later this proportion had grown to 15% [25], and even fewer guidelines provided information on how the COI had been managed. The proportion of guidelines that disclose COI of panel members has been shown to have further increased from 2006 to 2016 [26][27]. These data are in line with the finding of the present study. Reporting of COI of panel members of guidelines in anaesthesiology has increased from 28% to about 87% during the last 12 years. Up to now, however, very little research has been performed to examine how “informative” disclosures were. Hilda Bastian, in 2016, highlighted the lack of clarity of COI disclosures in reports of clinical trials and underlined that the visibility of COI disclosures needed to be improved [26]. Alhazzani et al stressed that intellectual and institutional COI were still largely overlooked during guideline development [2]. The problem regarding the lack of attention given to the impact of sponsors and authors’ COI has been previously highlighted in the context of systematic reviews [28].

### What does this study add?

The present study is the first to examine, from a reader’s point of view, the accessibility, transparency and clarity of information that can be derived from reporting of COI in guidelines in anaesthesia. COI are not systematically reported and when reported, statements very rarely provide a clear description of what the nature of the conflict was, and how it could have influenced the recommendations. Also, guidelines still rarely report on how COI were managed. Whether they were managed, but their management not reported, or not managed at all, remains unclear. We also show that although the reporting rate of COI has increased over the years, there is still room for improvement. For example, we were quite surprised by the diversity of the labels of the COI disclosures, even in one given journal. We suggest that COI disclosures should be presented in a separate paragraph, clearly identified as “conflicts of interest disclosures”. This work may be a basis for future recommendations on how COI ought to be reported in guidelines.

### Strengths and limitations

One of the strengths of our study is that we examined systematically searched guidelines across ten major anaesthesiology journals over a 12-year period, regardless of the topic. Two authors extracted the data independently, to minimise the risk of data extraction errors and inconsistencies.

One limitation of our study is that we did not check for the veracity of the disclosures. About 25% of the guidelines reported that none of the panel members had any COI, which may not necessarily be true. In fact recent studies have suggested that authors often fail to report real COI [3][29][30]. Also, we have not attempted to differentiate between financial COI (sometimes called direct COI) and non-financial COI (sometimes called indirect COI or academic COI). It has been shown that non-financial COI are frequent and may be as important as financial ones [11]. We have not performed subgroup analyses according to the type of sponsorship (industry or medical societies). As medical societies may receive sponsorship from industry, the distinction becomes debatable [31]. We cannot exclude the possibility that we have missed a guideline. Interestingly, we realised that when searching for guidelines using the filter “guideline” in PubMed, some guidelines we knew of could not be identified. It remains unclear on which basis recommendations are indexed as “guidelines” in electronic databases. Some reports included in our analyses may not be regarded by everyone as “real” guidelines since some looked more like short reports of expert opinions. However, we included these reports on a consistency basis; all were published with a sub-heading “guideline” or the term “guideline” or “consensus statement” was in the title.

### Where do we go from here—research agenda

It may be hypothesised that in the specific context of clinical practice guidelines, COI influence the direction or strength of recommendations [11]. However, this has never been formally demonstrated in the field of anaesthesia. Future research should examine the impact of COI of panel members, or of chairpersons, on the guideline recommendations. Also, recent concerns have focussed on non-financial COI [32]. It has been suggested that those are more likely to arise when panel members are specialists in the area under review, since they may have a strong opinion on the topic and may influence their peers [33]. The relative importance of both financial and non-financial COI requires further information [2]. Responsibility is an important issue that was highlighted in other settings and that needs further clarification [34][35]. Who should check for veracity, transparency and clarity of COI disclosures in guidelines? It has been suggested that journals should provide more detailed information on which COI need to be declared, and that authors must be warned about potential sanctions for those who fail to comply [36]. Others have suggested that the education of authors and reviewers should be improved [37], or that the chairpersons should be better trained on how to manage COI [23]. A more extreme view would be to refuse to publish guidelines where panellists have COI or when a description of how COI have been managed is lacking [26].

## Conclusion

In guidelines in anaesthesiology, COI of panel members or chairpersons, and the potential impact of sponsors of the guideline, are still not regularly reported. When they are reported, they are often not explicit enough to enable readers to understand what the nature of the COI or the impact of the sponsor of the guideline was, and how it could have influenced the recommendations. Finally, a description of the management of COI of panellists and of the potential influence of sponsors is often missing. Recommendations are needed to solve these issues.

## Supporting information

S1 Table Analysed guidelines. List of abbreviations:
na: not applicable
Description of terms:
°unclear 1: unclear whether description of how COI were managed needed or not (no COI nor sponsorship disclosed)
°unclear 2: unclear whether description of how COI were managed were needed or not since not all potential COI reported (panelist + chair or sponsor or none
All guidelines' references are available on S1 References.
(PDF)

S2 Table Reporting of disclosed COI and sponsor according to year of publication
List of abbreviations:
COI: conflict of interest
Description of terms:
*under the title or subtitle
(1): reported lack of COI of chair and panelist and no sponsor
(2): although panel members declared not having COI, there was a lack of disclosure of a sponsor or the chairperson could not be identified
(3): at least one COI or sponsorship disclosed
p1: p-value from the chi2 test
p2: p-value for linear trend over time.
(PDF)

S1 References References of the included guidelines. (PDF)
