# Supplementary material for: Reporting of conflicts of interest and of sponsorship of guidelines in anaesthesiology. A cross-sectional study
Source: PLoS One. 2019 Feb 27;14(2):e0212327. doi: 10.1371/journal.pone.0212327 (PMC6392260; doi:10.1371/journal.pone.0212327)
Supplement: S1 Table — List of abbreviations: na: not applicable Description of terms: °unclear 1: unclear whether description of how COI were managed needed or not (no COI nor sponsorship disclosed) °unclear 2: unclear whether description of how COI were managed were needed or not since not all potential COI reported (panelist + chair or sponsor or none All guidelines' references are available on S1 References. (PDF) [file pone.0212327.s001.pdf]

S1 Table

Id	Year	Article	Reporting of COI disclosures in the published report	Format of the disclosure	COI of panel members	Chairperson identifiable	COI of Chairperson	Sponsor of the guideline	Type of sponsoring	Need for COI's management	COI's management	Number of panellists
Anaesthesia												
A1	2008	Infection control in anaesthesia	Not reported	na	na	yes	na	Not reported	na	Unclear 1°	None reported	6
A2	2009	Suspected anaphylactic reactions associated with anaesthesia	Not reported	na	na	yes	na	Not reported	na	Unclear 1°	None reported	16
A3	2010	Blood transfusion and the anaesthetist: management of massive haemorrhage	Not reported	na	na	yes	na	Not reported	na	Unclear 1°	None reported	10
A4	2010	Safety in magnetic resonance units: an update	Not reported	na	na	yes	na	Not reported	na	Unclear 1°	None reported	12
A5	2011	Day case and short stay surgery: 2	Not reported	na	na	yes	na	Not reported	na	Unclear 1°	None reported	11
A6	2012	Multidisciplinary guidelines for the management of tracheostomy and laryngectomy airway emergencies	Not reported	na	na	no	na	Reported with description	Medical society	Measures clearly needed since COI disclosed	None reported	4
A7	2012	Checking anaesthetic equipment 2012: association of anaesthetists of Great Britain and Ireland	Not reported	na	na	yes	na	Not reported	na	Unclear 1°	None reported	9
A8	2012	Difficult Airway Society Guidelines for the management of tracheal extubation	Published	Reported in a separate paragraph containing the term "interest"	Reported without description	yes	Reported without description	Not reported	na	Measures clearly needed since COI disclosed	None reported	6
A9	2012	Management of proximal femoral fractures 2011: Association of Anaesthetists of Great Britain and Ireland	Published	Reported in a separate paragraph containing the term "interest"	Reported without description	yes	Reported without description	Not reported	na	Measures clearly needed since COI disclosed	None reported	12
A10	2013	Regional anaesthesia and patients with abnormalities of coagulation	Not reported	na	na	no	na	Not reported	na	Unclear 1°	None reported	11
A11	2013	Immediate post-anaesthesia recovery 2013: Association of Anaesthetists of Great Britain and Ireland	Published	Reported in a separate paragraph containing the term "interest"	Reported without description	yes	Reported without description	Reported that there was none	none	Measures clearly needed since COI disclosed	None reported	9
A12	2014	Safety guideline: skin antisepsis for central neuraxial blockade	Published	Reported in a separate paragraph containing the term "interest"	Reported with description	no	na	Reported that there was none	none	Measures clearly needed since COI disclosed	Measures have been taken with description	7
A13	2014	Arterial line blood sampling: preventing hypoglycaemic brain injury 2014: the Association of Anaesthetists of Great Britain and Ireland	Published	Reported in a separate paragraph containing the term "interest"	Reported that they were none	no	na	Reported that there was none	none	Unclear 2°	None reported	4
A14	2014	Peri-operative care of the elderly 2014: Association of Anaesthetists of Great Britain and Ireland	Published	Reported in a separate paragraph containing the term "interest"	Reported without description	yes	Reported without description	Not reported	na	Measures clearly needed since COI disclosed	None reported	11
A15	2015	Obstetric Anaesthetists : Association and Difficult Airway Society	Published	Reported in a separate paragraph containing the term "interest"	Reported without description	yes	na	Reported without description	Medical society	Measures clearly needed since COI disclosed	Not convincing measure taken	7
A16	2015	Peri-operative management of the obese surgical patient 2015	Not reported	na	na	yes	na	Not reported	na	Unclear 1°	None reported	15

S1 Table

Id	Year	Article	Reporting of COI disclosures in the published report	Format of the disclosure	COI of panel members	Chairperson identifiable	COI of Chairperson	Sponsor of the guideline	Type of sponsoring	Need for COI's management	COI's management	Number of panellists
A17	2015	Safety guideline: reducing the risk from cemented hemiarthroplasty for hip fracture 2015	Not reported	na	na	no	na	Not reported	na	Unclear 1°	None reported	9
A18	2016	Safe vascular access 2016	Published	Reported in a separate paragraph without the term "interest"	Reported without description	yes	Reported without description	Not reported	na	Measures clearly needed since COI disclosed	None reported	11
A19	2016	The measurement of adult blood pressure and management of hypertension before elective surgery	Not reported	na	na	no	na	Not reported	na	Unclear 1°	None reported	8
A20	2016	AAGBI guidelines: the use of blood components and their alternatives 2016	Published	Reported in a separate paragraph without the term "interest"	Reported without description	yes	Reported without description	Not reported	na	Measures clearly needed since COI disclosed	None reported	14
A21	2017	International consensus statement on the peri-operative management of anaemia and iron deficiency	Published	Reported in a separate paragraph without the term "interest"	Reported without description	yes	Reported without description	Reported and described	Industry	Measures clearly needed since COI disclosed	None reported	15
Anesthesia & Analgesia												
B1	2007	Society for Ambulatory Anesthesia guidelines for the management of postoperative nausea and vomiting	Not reported	na	na	no	na	Not reported	na	Unclear 1°	None reported	17
B2	2007	Guidelines for performing a comprehensive epicardial echocardiography examination: recommendations of the American Society of Echocardiography and the Society of Cardiovascular Anesthesiologists	Not reported	na	na	no	na	Not reported	na	Unclear 1°	None reported	7
B3	2008	Guidelines for the performance of a comprehensive intraoperative epiaortic ultrasonographic examination: recommendations of the American Society of Echocardiography and the Society of Cardiovascular Anesthesiologists; endorsed by the Society of Thoracic Surgeons	Not reported	na	na	yes	na	Not reported	na	Unclear 1°	None reported	8
B4	2008	ACC/AHA 2007 guidelines on perioperative cardiovascular evaluation and care for noncardiac surgery: executive summary: a report of the American College of Cardiology/American Heart Association Task Force on Practice Guidelines (Writing Committee to Revise the 2002 Guidelines on Perioperative Cardiovascular Evaluation for Noncardiac Surgery)	Published	Reported in a separate paragraph without the term "interest"	Reported without description	yes	Reported that they were none	Not reported	na	Measures clearly needed since COI disclosed	None reported	12
B5	2009	Consensus statement: First International Workshop on Anesthetics and Alzheimer's disease	Not reported	na	na	no	na	Not reported	na	Unclear 1°	None reported	16
B6	2010	Society for Ambulatory Anesthesia consensus statement on perioperative blood glucose management in diabetic patients undergoing ambulatory surgery	Not reported	na	na	no	na	Not reported	na	Unclear 1°	None reported	8
B7	2010	2010 ACCF/AHA/AATS/ACR/ASA/SCA/SCAI/SIR/STS/SVM Guidelines for the diagnosis and management of patients with thoracic aortic disease: Executive summary:	Published	Reported in a separate paragraph without the term "interest"	Reported without description	yes	Reported without description	Not reported	na	Measures clearly needed since COI disclosed	None reported	18
B8	2012	Special articles: guidelines for performing ultrasound guided vascular cannulation: recommendations of the American Society of Echocardiography and the Society Of Cardiovascular Anesthesiologists	Published	Not reported in a separate paragraph	Reported that they were none	no	na	Not reported	na	Unclear 2°	None reported	7

S1 Table

Id	Year	Article	Reporting of COI disclosures in the published report	Format of the disclosure	COI of panel members	Chairperson identifiable	COI of Chairperson	Sponsor of the guideline	Type of sponsoring	Need for COI's management	COI's management	Number of panellists
B9	2012	Special Articles: 2011 ACCF/AHA Guideline for Coronary Artery Bypass Graft Surgery: executive summary: a report of the American College of Cardiology Foundation/American Heart Association Task Force on Practice Guidelines	Published	Reported in a separate paragraph without the term "interest"	Reported without description	yes	Reported that they were none	Not reported	na	Measures clearly needed since COI disclosed	Measures have been taken with description	23
B10	2014	The Society for Obstetric Anesthesia and Perinatology consensus statement on the management of cardiac arrest in pregnancy	Published	Reported in a separate paragraph without the term "interest"	Reported without description	yes	Reported that they were none	Reported that there was none	None	Measures clearly needed since COI disclosed	None reported	16
B11	2014	Consensus guidelines for the management of postoperative nausea and vomiting	Published	Reported in a separate paragraph without the term "interest"	Reported without description	no	na	Reported that there was none	None	Measures clearly needed since COI disclosed	None reported	20
B12	2014	Guidelines for performing a comprehensive transesophageal echocardiographic examination: recommendations from the American Society of Echocardiography and the Society of Cardiovascular Anesthesiologists	Published	Not reported in a separate paragraph	Reported without description	yes	Reported that they were none	Not reported	na	Measures clearly needed since COI disclosed	None reported	11
B13	2011	Guidelines for pediatric perioperative care during short-term plastic reconstructive surgical projects in less developed nations	Published	Not reported in a separate paragraph	Reported that they were none	no	na	Not reported	na	Unclear 2°	None reported	8
B14	2012	Society for Ambulatory Anesthesia consensus statement on preoperative selection of adult patients with obstructive sleep apnea scheduled for ambulatory surgery	Published	Reported in a separate paragraph without the term "interest"	Reported without description	yes	Reported that they were none	Reported that there was none	None	measures clearly needed since COI disclosed	None reported	4
B15	2013	Special article: basic perioperative transesophageal echocardiography examination: a consensus statement of the American Society of Echocardiography and the Society of Cardiovascular Anesthesiologists	Published	Not reported in a separate paragraph	Reported without description	yes	Reported without description	Not reported	na	measures clearly needed since COI disclosed	None reported	10
B16	2016	Society of Anesthesia and Sleep Medicine Guidelines on Preoperative Screening and Assessment of Adult Patients With Obstructive Sleep Apnea	Published	Reported in a separate paragraph without the term "interest"	Reported without description	no	na	Reported that there was none	None	measures clearly needed since COI disclosed	None reported	28
B17	2016	SCAI/CCAS/SPA Expert Consensus Statement for Anesthesia and Sedation Practice: Recommendations for Patients Undergoing Diagnostic and Therapeutic Procedures in the Pediatric and Congenital Cardiac Catheterization Laboratory	Published	Reported in a separate paragraph without the term "interest"	Reported that they were none	no	na	Not reported	na	Unclear 2°	None reported	12
B18	2018	The Society of Thoracic Surgeons, The Society of Cardiovascular Anesthesiologists, and The American Society of ExtraCorporeal Technology: Clinical Practice Guidelines-Anticoagulation During Cardiopulmonary Bypass	Not reported	na	na	no	na	Not reported	na	Unclear 1°	None reported	7
B19	2018	Consensus Statement by the Congenital Cardiac Anesthesia Society: Milestones for the Pediatric Cardiac Anesthesia Fellowship	Published	Not reported in a separate paragraph	Reported that they were none	no	na	Not reported	na	Unclear 2°	None reported	9
B20	2018	Society of Anesthesia and Sleep Medicine Guideline on Intraoperative Management of Adult Patients With Obstructive Sleep Apnea	Published	Reported in a separate paragraph without the term "interest"	Reported without description	no	na	Reported that there was none	na	Measures clearly needed since COI disclosed	None reported	14
B21	2018	American Society for Enhanced Recovery and Perioperative Quality Initiative Joint Consensus Statement on Patient-Reported Outcomes in an Enhanced Recovery Pathway	Published	Reported in a separate paragraph without the term "interest"	Reported without description	yes	Reported without description	Reported without description	Medical society	Measures clearly needed since COI disclosed	None reported	25

S1 Table

Id	Year	Article	Reporting of COI disclosures in the published report	Format of the disclosure	COI of panel members	Chairperson identifiable	COI of Chairperson	Sponsor of the guideline	Type of sponsoring	Need for COI's management	COI's management	Number of panellists
B22	2018	The Society for Obstetric Anesthesia and Perinatology Consensus Statement on the Anesthetic Management of Pregnant and Postpartum Women Receiving Thromboprophylaxis or Higher Dose Anticoagulants	Published	Not reported in a separate paragraph	Reported that they were none	yes	Reported that they were none	Reported that there was none	na	No preventive measures needed	None reported	9
Anesthesiology												
C1	2010	Practice guidelines for perioperative transesophageal echocardiography An updated report by the American Society of Anesthesiologists and the Society of Cardiovascular Anesthesiologists Task Force on Transesophageal Echocardiography	Not reported	na	na	yes	na	Not reported	na	Unclear 1°	None reported	13
C2	2010	Practice guidelines for chronic pain management: an updated report by the American Society of Anesthesiologists Task Force on Chronic Pain Management and the American Society of Regional Anesthesia and Pain Medicine	Not reported	na	na	yes	na	Not reported	na	Unclear 1°	None reported	12
C3	2012	Practice guidelines for central venous access: a report by the American Society of Anesthesiologists Task Force on Central Venous Access	Not reported	na	na	yes	na	Not reported	na	Unclear 1°	None reported	12
C4	2012	Practice guidelines for acute pain management in the perioperative setting: an updated report by the American Society of Anesthesiologists Task Force on Acute Pain Management	Not reported	na	na	yes	na	Not reported	na	Unclear 1°	None reported	5
C5	2013	Practice guidelines for postanesthetic care: an updated report by the American Society of Anesthesiologists Task Force on Postanesthetic Care	Not reported	na	na	yes	na	Not reported	na	Unclear 1°	None reported	8
C6	2013	Practice guidelines for management of the difficult airway: an updated report by the American Society of Anesthesiologists Task Force on Management of the Difficult Airway	Not reported	na	na	yes	na	Not reported	na	Unclear 1°	None reported	6
C7	2014	Practice guidelines for the perioperative management of patients with obstructive sleep apnea: an updated report by the American Society of Anesthesiologists Task Force on Perioperative Management of patients with obstructive sleep apnea	Published	Reported in a separate paragraph containing the term "interest"	Reported that they were none	yes	Reported that they were none	Reported without description	Medical society	Measures clearly needed since COI disclosed	None reported	9
C8	2015	Practice Guidelines for Perioperative Blood Management An Updated Report by the American Society of Anesthesiologists Task Force on Perioperative Blood Management	Published	Reported in a separate paragraph containing the term "interest"	Reported that they were none	yes	Reported that they were none	Reported without description	Medical society	Measures clearly needed since COI disclosed	None reported	10
C9	2016	An Updated Report by the American Society of Anesthesiologists Task Force on Obstetric Anesthesia and the Society for Obstetric Anesthesia and Perinatology	Published	Reported in a separate paragraph containing the term "interest"	Reported that they were none	yes	Reported that they were none	Reported without description	Academic or institutional	Measures clearly needed since COI disclosed	None reported	11
C10	2016	Practice Guidelines for the Prevention, Detection, and Management of Respiratory Depression Associated with Neuraxial Opioid Administration: An Updated Report by the American Society of Anesthesiologists Task Force on Neuraxial Opioids and the American Society of Regional Anesthesia and Pain Medicine	Published	Reported in a separate paragraph containing the term "interest"	Reported that they were none	yes	Reported that they were none	Reported without description	Academic or institutional	measures clearly needed since COI disclosed	None reported	10

S1 Table

Id	Year	Article	Reporting of COI disclosures in the published report	Format of the disclosure	COI of panel members	Chairperson identifiable	COI of Chairperson	Sponsor of the guideline	Type of sponsoring	Need for COI's management	COI's management	Number of panellists
C11	2017	Practice Guidelines for Preoperative Fasting and the Use of Pharmacologic Agents to Reduce the Risk of Pulmonary Aspiration: Application to Healthy Patients Undergoing Elective Procedures: An Updated Report by the American Society of Anesthesiologists Task Force on Preoperative Fasting and the Use of Pharmacologic Agents to Reduce the Risk of Pulmonary Aspiration	Published	Reported in a separate paragraph containing the term "interest"	na	yes	Reported that they were none	Reported without description	Academic or institutional	Measures clearly needed since COI disclosed	None reported	6
C12	2018	Practice Guidelines for Moderate Procedural Sedation and Analgesia 2018: A Report by the American Society of Anesthesiologists Task Force on Moderate Procedural Sedation and Analgesia, the American Association of Oral and Maxillofacial Surgeons, American College of Radiology, American Dental Association, American Society of Dentist Anesthesiologists, and Society of Interventional Radiology	Published	Reported in a separate paragraph containing the term "interest"	na	yes	Reported that they were none	Reported without description	Medical society	Measures clearly needed since COI disclosed	Not convincing measure taken	13
British Journal of Anaesthesia												
D1	2010	Recognizing and managing a malignant hyperthermia crisis: guidelines from the European Malignant Hyperthermia Group	Published	Reported in a separate paragraph containing the term "interest"	Reported that they were none	no	na	Not reported	na	Unclear 2°	None reported	7
D2	2011	Detection, evaluation, and management of preoperative anaemia in the elective orthopaedic surgical patient: NATA guidelines	Published	Reported in a separate paragraph containing the term "interest"	Reported without description	no	na	Reported without description	Mix of funding	Unclear 1°	None reported	13
D3	2013	Low back and radicular pain: a pathway for care developed by the British Pain Society	Published	Reported in a separate paragraph containing the term "interest"	Reported without description	no	na	Not reported	na	Measures clearly needed since COI disclosed	None reported	4
D4	2015	European Malignant Hyperthermia Group guidelines for investigation of malignant hyperthermia susceptibility	Published	Reported in a separate paragraph containing the term "interest"	Reported without description	no	na	Not reported	na	Measures clearly needed since COI disclosed	None reported	8
D5	2008	Perioperative anaemia management: consensus statement on the role of intravenous iron	Published	Reported in a separate paragraph without the term "interest"	Reported without description	no	na	Reported and described	Industry	measures clearly needed since COI disclosed	None reported	6
D6	2013	Differential diagnosis of facial pain and guidelines for management	Published	Reported in a separate paragraph containing the term "interest"	na	no	na	Reported without description	Industry	measures clearly needed since COI disclosed	None reported	1
D7	2015	Difficult Airway Society 2015 guidelines for management of unanticipated difficult intubation in adults	Published	Reported in a separate paragraph containing the term "interest"	Reported without description	no	na	Reported without description	Medical society	measures clearly needed since COI disclosed	None reported	9
D8	2015	Consensus statement from the BJA Workshop on Cancer and Anaesthesia	Published	Reported in a separate paragraph containing the term "interest"	Reported without description	no	na	Not reported	na	measures clearly needed since COI disclosed	None reported	19

S1 Table

Id	Year	Article	Reporting of COI disclosures in the published report	Format of the disclosure	COI of panel members	Chairperson identifiable	COI of Chairperson	Sponsor of the guideline	Type of sponsoring	Need for COI's management	COI's management	Number of panellists
D9	2018	Guidelines for the management of tracheal intubation in critically ill adults	Published	Reported in a separate paragraph containing the term "interest"	Reported without description	yes	Reported without description	Reported without description	Medical society	Measures clearly needed since COI disclosed	None reported	7
D10	2018	Management of perceived devastating brain injury after hospital admission: a consensus statement from stakeholder professional organizations	Published	Reported in a separate paragraph containing the term "interest"	Reported that they were none	yes	Reported that they were none	Not reported	na	Unclear 2°	None reported	7
D11	2018	Perioperative cardiopulmonary exercise testing (CPET): consensus clinical guidelines on indications, organization, conduct, and physiological interpretation	Published	Reported in a separate paragraph containing the term "interest"	Reported without description	no	na	Reported and described	Academic or institutional	Measures clearly needed since COI disclosed	None reported	11
Canadian Journal of Anesthesia												
F1	2017	CCCS-SSAI WikiRecs Clinical Practice Guideline: vasopressor blood pressure targets in critically ill adults with hypotension	Published	Reported in a separate paragraph containing the term "interest"	Reported that they were none	yes	Reported that they were none	Not reported	na	Unclear 2°	None reported	13
F2	2018	Guidelines to the Practice of Anesthesia - Revised Edition 2018	Published	Reported in a separate paragraph containing the term "interest"	Reported that they were none	yes	Reported that they were none	Not reported	na	Unclear 2°	None reported	9
European Journal of Anaesthesiology												
E1	2007	Guidelines for anaesthesiologist specialist training in pain medicine	Not reported	na	na	no	na	Not reported	na	Unclear 1°	None reported	8
E2	2007	Guidelines for sedation and/or analgesia by non-anaesthesiology doctors	Not reported	na	na	no	na	Not reported	na	Unclear 1°	None reported	7
E3	2007	Guidelines for safety and quality in anaesthesia practice in the European Union	Not reported	na	na	no	na	Not reported	na	Unclear 1°	None reported	7
E4	2007	Charter on continuing medical education/continuing professional development approved by the UEMS Specialist Section and European Board of Anaesthesiology	Not reported	na	na	no	na	Not reported	na	Unclear 1°	None reported	4
E5	2007	Core curriculum in emergency medicine integrated in the specialty of anaesthesiology	Not reported	na	na	no	na	Not reported	na	Unclear 1°	None reported	4
E6	2008	Education and training in anaesthesia--revised guidelines by the European Board of Anaesthesiology, Reanimation and Intensive Care	Not reported	na	na	no	na	Not reported	na	Unclear 1°	None reported	6
E7	2009	Quality and safety guidelines of post anaesthesia care	Not reported	na	na	no	na	Not reported	na	Unclear 1°	None reported	3
E8	2010	European Society of Gastrointestinal Endoscopy, European Society of Gastroenterology and Endoscopy Nurses and Associates, and the European Society of Anaesthesiology Guideline: Non-anaesthesiologist administration of propofol for GI endoscopy	Published	Reported in a separate paragraph without the term "interest"	Reported without description	no	na	Reported without description	Medical society	Measures clearly needed since COI disclosed	None reported	14
E9	2010	Regional anaesthesia and antithrombotic agents: recommendations of the European Society of Anaesthesiology	Published	Reported in a separate paragraph without the term "interest"	Reported without description	no	na	Not reported	na	Measures clearly needed since COI disclosed	None reported	6

S1 Table

Id	Year	Article	Reporting of COI disclosures in the published report	Format of the disclosure	COI of panel members	Chairperson identifiable	COI of Chairperson	Sponsor of the guideline	Type of sponsoring	Need for COI's management	COI's management	Number of panellists
E10	2010	Guidelines for pre-operative cardiac risk assessment and perioperative cardiac management in non-cardiac surgery	Online only	na	na	yes	na	Reported without description	Medical society	Measures clearly needed since COI disclosed	None reported	22
E11	2011	Perioperative fasting in adults and children: guidelines from the European Society of Anaesthesiology	Published	Reported in a separate paragraph without the term "interest"	Reported without description	no	na	Not reported	na	Measures clearly needed since COI disclosed	None reported	8
E12	2011	Preoperative evaluation of the adult patient undergoing non-cardiac surgery: guidelines from the European Society of Anaesthesiology	Published	Reported in a separate paragraph without the term "interest"	Reported without description	no	na	Not reported	na	Measures clearly needed since COI disclosed	None reported	10
E13	2011	European consensus statement for intraoperative fluid therapy in children	Published	Reported in a separate paragraph without the term "interest"	Reported that they were none	no	na	Not reported	na	Unclear 2°	None reported	7
E14	2013	Pre-interventional haemostatic assessment: Guidelines from the French Society of Anaesthesia and Intensive Care	Published	Reported in a separate paragraph without the term "interest"	Reported without description	no	na	Reported without description	Medical society	Measures clearly needed since COI disclosed	None reported	5
E15	2014	2014ESC/ESA Guidelines on non-cardiacsurgery:cardio vascular assessment and management	Online only	na	na	yes	na	Reported without description	Medical society	Measures clearly needed since COI disclosed	None reported	24
E16	2015	Standards for definitions and use of outcome measures for clinical effectiveness research in perioperative medicine: European Perioperative Clinical Outcome (EPCO) definitions	Published	Reported in a separate paragraph without the term "interest"	Reported without description	yes	Reported without description	Reported without description	Medical society	Measures clearly needed since COI disclosed	None reported	12
E17	2011	Non-anaesthesiologists should not be allowed to administer propofol for procedural sedation: a Consensus Statement of 21 European National Societies of Anaesthesia	Not reported	na	na	no	na	Not reported	na	Unclear 1°	None reported	1
E18	2016	Intravascular volume therapy in adults: Guidelines from the Association of the Scientific Medical Societies in Germany	Online only	na	na	no	na	Reported without description	Medical society	measures clearly needed since COI disclosed	Measures have been taken with description	20
E19	2017	European Society of Anaesthesiology evidence-based and consensus-based guideline on postoperative delirium	Online only	Reported in a separate paragraph without the term "interest"	Reported with description	yes	Reported and described	Reported without description	Mix of funding	measures clearly needed since COI disclosed	Measures have been taken with description	18
E20	2017	Management of severe perioperative bleeding: guidelines from the European Society of Anaesthesiology: First update 2016	Online only	Reported in a separate paragraph without the term "interest"	Reported without description	yes	Reported without description	Reported without description	Medical society	measures clearly needed since COI disclosed	None reported	25
E21	2018	European guidelines on perioperative venous thromboembolism prophylaxis	Online only	Reported in a separate paragraph without the term "interest"	Reported without description	no	na	Reported without description	Medical society	measures clearly needed since COI disclosed	None reported	1
E22	2018	European Society of Anaesthesiology and European Board of Anaesthesiology guidelines for procedural sedation and analgesia in adults	Online only	Reported in a separate paragraph without the term "interest"	Reported without description	yes	Reported without description	Reported that there was none	None	measures clearly needed since COI disclosed	None reported	13

S1 Table

Id	Year	Article	Reporting of COI disclosures in the published report	Format of the disclosure	COI of panel members	Chairperson identifiable	COI of Chairperson	Sponsor of the guideline	Type of sponsoring	Need for COI's management	COI's management	Number of panellists
E23	2018	Pre-operative evaluation of adults undergoing elective noncardiac surgery: Updated guideline from the European Society of Anaesthesiology	Online only	Reported in a separate paragraph without the term "interest"	Reported that they were none	yes	Reported that they were none	Reported without description	Medical society	measures clearly needed since COI disclosed	None reported	23
Journal of Neurosurgical Anesthesiology												
G1	2013	Neuroanesthesiology fellowship training: curricular guidelines from the Society for Neuroscience in Anesthesiology and Critical Care	Published	Not reported in a separate paragraph	Reported that they were none	no	na	Reported that there was none	None	No preventive measures needed	None reported	7
G2	2017	Perioperative Management of Adult Patients With External Ventricular and Lumbar Drains: Guidelines From the Society for Neuroscience in Anesthesiology and Critical Care	Published	Not reported in a separate paragraph	Reported without description	no	na	Not reported	na	Measures clearly needed since COI disclosed	None reported	10
G3	2014	Society for Neuroscience in Anesthesiology and Critical Care Expert consensus statement: anesthetic management of endovascular treatment for acute ischemic stroke*: endorsed by the Society of NeuroInterventional Surgery and the Neurocritical Care Society	Published	Not reported in a separate paragraph	Reported that they were none	no	na	Not reported	na	Unclear 2°	None reported	6
G4	2014	Perioperative care of patients at high risk for stroke during or after non-cardiac, non-neurologic surgery: consensus statement from the Society for Neuroscience in Anesthesiology and Critical Care*	Published	Not reported in a separate paragraph	Reported that they were none	no	na	Reported without description	Academic or institutional	Measures clearly needed since COI disclosed	None reported	5
Minerva Anestesiologica												
H1	2008	Treatment of minor and severe traumatic brain injury National reference guidelines	Published	Not reported in a separate paragraph	Reported that they were none	no	na	Not reported	na	Unclear 2°	None reported	24
H2	2013	II Italian intersociety consensus statement on antithrombotic prophylaxis in orthopedics and traumatology	Published	Not reported in a separate paragraph	Reported without description	no	na	Not reported	na	Measures clearly needed since COI disclosed	None reported	9
H3	2007	SIAARTI - IRC recommendations for organizing responses to In-Hospital emergencies	Published	Not reported in a separate paragraph	Reported that they were none	no	na	Not reported	na	Unclear 2°	None reported	10
H4	2009	Recommendations for airway control and difficult airway management in thoracic anesthesia and lung separation procedures	Not reported	na	na	no	na	Not reported	na	Unclear 1°	None reported	7
H5	2010	Postoperative pain treatment SIAARTI Recommendations 2010 Short version	Published	Reported in a separate paragraph without the term "interest"	Reported without description	no	na	Not reported	na	Unclear 1°	None reported	34
H6	2014	Preoperative evaluation in infants and children: recommendations of the Italian Society of Pediatric and Neonatal Anesthesia and Intensive Care (SARNePI)	Published	Not reported in a separate paragraph	Reported that they were none	no	na	Not reported	na	Unclear 2°	None reported	23
H7	2009	Perioperative antibiotic prophylaxis in adults Outline of the principal recommendations National reference guidelines	Not reported	na	na	no	na	Not reported	na	Unclear 1°	None reported	19
H8	2011	Italian intersociety consensus statement on antithrombotic prophylaxis in hip and knee replacement and in femoral neck fracture surgery	Not reported	na	na	no	na	Not reported	na	Unclear 1°	None reported	8
Regional Anesthesia and Pain Medicine												

S1 Table

Id	Year	Article	Reporting of COI disclosures in the published report	Format of the disclosure	COI of panel members	Chairperson identifiable	COI of Chairperson	Sponsor of the guideline	Type of sponsoring	Need for COI's management	COI's management	Number of panellists
I1	2010	Regional anesthesia in the patient receiving antithrombotic or thrombolytic therapy: American Society of Regional Anesthesia and Pain Medicine Evidence-Based Guidelines (Third Edition)	Not reported	na	na	no	na	Not reported	na	Unclear 1°	None reported	12
I2	2015	Interventional spine and pain procedures in patients on antiplatelet and anticoagulant medications: guidelines from the American Society of Regional Anesthesia and Pain Medicine, the European Society of Regional Anaesthesia and Pain Therapy, the American Academy of Pain Medicine, the International Neuromodulation Society, the North American Neuromodulation Society, and the World Institute of Pain	Published	Not reported in a separate paragraph	Reported that they were none	no	na	Not reported	na	Unclear 2°	None reported	8
I3	2015	Guidelines for fellowship training in regional anesthesiology and acute pain medicine: third edition, 2014	Published	Not reported in a separate paragraph	Reported that they were none	no	na	Not reported	na	Unclear 2°	None reported	7
I4	2009	The American Society of Regional Anesthesia and Pain Medicine and the European Society Of Regional Anaesthesia and Pain Therapy Joint Committee recommendations for education and training in ultrasound-guided regional anesthesia	Published	Reported in a separate paragraph without the term "interest"	Reported without description	no	na	Reported without description	Medical society	Measures clearly needed since COI disclosed	None reported	7
I5	2012	The American Society of Regional Anesthesia and Pain Medicine, the European Society of Regional Anaesthesia and Pain Therapy, and the Asian Australasian Federation of Pain Societies Joint Committee recommendations for education and training in ultrasound-guided interventional pain procedures	Not reported	na	na	no	na	Not reported	na	Unclear 1°	None reported	7
I6	2018	Interventional Spine and Pain Procedures in Patients on Antiplatelet and Anticoagulant Medications (Second Edition): Guidelines From the American Society of Regional Anesthesia and Pain Medicine, the European Society of Regional Anaesthesia and Pain Therapy, the American Academy of Pain Medicine, the International Neuromodulation Society, the North American Neuromodulation Society, and the World Institute of Pain	Published	Not reported in a separate paragraph	Reported without description	no	na	Not reported	na	Measures clearly needed since COI disclosed	None reported	8
I7	2018	Regional Anesthesia in the Patient Receiving Antithrombotic or Thrombolytic Therapy: American Society of Regional Anesthesia and Pain Medicine Evidence-Based Guidelines (Fourth Edition)	Published	Not reported in a separate paragraph	Reported that they were none	no	na	Not reported	na	Unclear 2°	None reported	6

na: not applicable

unclear 1°: unclear whether specific procedures to minimise the risk of bias due to COIs or sponsors were needed or not (no disclosure of COI nor sponsorship)

unclear 2°: unclear whether procedures were needed or not since information was lacking (not all potential COI were reported [panelist + chair] and/or no information on the presence or absence of a sponsor)
