# Supplementary material for: Reporting of conflicts of interest and of sponsorship of guidelines in anaesthesiology. A cross-sectional study
Source: PLoS One. 2019 Feb 27;14(2):e0212327. doi: 10.1371/journal.pone.0212327 (PMC6392260; doi:10.1371/journal.pone.0212327)
Supplement: S2 Table — Reporting of disclosed COI and sponsor according to year of publication List of abbreviations: COI: conflict of interest Description of terms: *under the title or subtitle (1): reported lack of COI of chair and panelist and no sponsor (2): although panel members declared not having COI, there was a lack of disclosure of a sponsor or the chairperson could not be identified (3): at least one COI or sponsorship disclosed p1: p-value from the chi2 test p2: p-value for linear trend over time. (PDF) [file pone.0212327.s002.pdf]

S2 Table. Reporting of disclosed COI and sponsors according to year of publication

	Total	2007-2010	2011-2014	2015-2018	p1	p2
**Number of analysed guidelines**						
**Journals**	**110**	32	39	39	0.169	
European Journal of Anaesthesiology	23	10	6	7		
Anesthesia & Analgesia	22	7	8	7		
Anaesthesia	21	4	10	7		
Anesthesiology	12	2	5	5		
British Journal of Anaesthesia	11	2	3	6		
Minerva Anestesiologica	8	5	3	0		
Regional Anesthesia and Pain Medicine	7	2	1	4		
Journal of Neurosurgical Anesthesiology	4	0	3	1		
Canadian Journal of Anesthesia	2	0	0	2		
International Journal of Obstetric Anesthesia	0	0	0	0		
**Reporting of COI disclosures**						
Accessibility of COI disclosures (n=110)						
Reported in the published guideline	70	9/32 (28%)	27/39 (69%)	34/39 (87%)		<0.001
Not reported in published guideline (published online or hidden behind access barriers)	40	23/32 (72%)	12/39 (31%)	5/39 (13%)		
Format of the published COI disclosures, when reported in the published guideline (n=70)					0.265	
Distinct paragraph including the term "interest"	25	1/9 (11%)	10/27 (37%)	14/34 (41%)		
Distinct paragraph, not clearly identified	27	6/9 (67%)	8/27 (30%)	13/34 (38%)		
No distinct paragraph*	18	2/9 (22%)	9/27 (33%)	7/34 (21%)		
Content of disclosure of COI of panel members, when reported in the published guideline (n=70)					0.874	
Reported lack of COI	27	3/9 (33%)	9/27 (33%)	15/34 (44%)		
Reported COI, with description of potential influence	2	0/9 (0%)	1/27 (4%)	1/34 (3%)		
Reported COI, without description of potential influence	41	6/9 (67%)	17/27 (63%)	18/34(53%)		
Content of disclosure of COI of chairperson, when reported in the published guideline (n=70)					0.593	
Reported lack of COI	16	1/9 (11%)	5/27(19%)	10/34 (29%)		
Reported COI, with description of potential influence	1	0/9 (0%)	0/27 (0%)	1/34 (3%)		
Reported COI, without description of potential influence	14	1/9 (11%)	5/27 (19%)	8/34 (24%)		
COI not reported	1	0/9 (0%)	0/27 (0%)	1/34 (3%)		
Chairperson not identified	38	7/9 (78%)	17/27 (63%)	14/34 (41%)		
**Sponsor of the guideline (n=110)**						
Reported in the published guideline	40	4/32 (13%)	15/39 (38%)	21/39 (54%)		0.001
Not reported in the published guideline	70	28/32 (88%)	24/39 (62%)	18/39 (46%)		
Content of disclosures of sponsor of guideline, when reported in the published guideline (n=40)						
Reported that there was none	12	0/4 (0%)	8/15 (53%)	4/21 (19%)	0.114	
Reported, with description of potential influence	4	1/4 (25%)	1/15 (7%)	2/21 (10%)		
Reported, without description of potential influence	24	3/4 (75%)	6/15 (40%)	15/21 (71%)		
Source of funding, when reported in the published guideline (n=28)					0.456	
Industry	3	1/4 (25%)	1/7 (14%)	1/17 (6%)		
Medical society	17	3/4 (75%)	3/7 (43%)	11/17 (65%)		
Academic/institutional	5	0/4 (0%)	1/7 (14%)	4/17 (24%)		
Mixed	3	0/4 (0%)	2/7 (29%)	1/17 (6%)		
**Description of the management of COI and sponsorship of the guideline**						
COI and/or sponsor disclosed (n=75)		10/32 (31%)	30/39 (77%)	35/39 (90)		<0.001
Management not needed [1]	3	0/10 (0%)	2/30 (7%)	1/35 (3%)	0.844	
Unclear whether management needed [2]	17	3/10 (30%)	6/30 (20%)	8/35 (23%)		
Management needed [3]	55	7/10 (70%)	22/30 (73%)	26/35 (74%)		
Management of COI described, when needed (n=55)						
Management of COI reported and described	6	0/7 (0%)	2/22 (9%)	4/26 (15%)	0.480	
No mention of management of COI	48	7/7 (100%)	20/22 (91%)	21/26 (85%)		

COI: conflict of interest; *below the title or subtitle; [1] reported lack of COI of chair and panelist and no sponsor; [2] although panel members declared not having COI, there was a lack of disclosure of a sponsor or the chairperson could not be identified; [3] at least one COI or sponsorship disclosed; p1: p-value from the chi2 test; p2: p-value for linear trend over time
