# Supplementary material for: Reporting of conflicts of interest and of sponsorship of guidelines in anaesthesiology. A cross-sectional study
Source: PLoS One. 2019 Feb 27;14(2):e0212327. doi: 10.1371/journal.pone.0212327 (PMC6392260; doi:10.1371/journal.pone.0212327)
Supplement: S1 References — (PDF) [file pone.0212327.s003.pdf]

S1 References

References of the included guidelines

A1 Infection Control in Anaesthesia: Infection control in anaesthesia. *Anaesthesia* **63,** 1027–1036 (2008).

A2 Membership of the Working Party *et al.* Suspected Anaphylactic Reactions Associated with Anaesthesia. *Anaesthesia* **64,** 199–211 (2009).

A3 Thomas, D. *et al.* Blood transfusion and the anaesthetist: management of massive haemorrhage: Blood transfusion and the anaesthetist: management of massive haemorrhage. *Anaesthesia* **65,** 1153–1161 (2010).

A4 Safety in magnetic resonance units: an update: Safety in magnetic resonance units. *Anaesthesia* **65,** 766–770 (2010).

A5 Day case and short stay surgery: 2: Guidelines: Day case and short stay surgery. *Anaesthesia* **66,** 417–434 (2011).

A6 McGrath, B. A., Bates, L., Atkinson, D. & Moore, J. A. Multidisciplinary guidelines for the management of tracheostomy and laryngectomy airway emergencies: Tracheostomy management guidelines. *Anaesthesia* **67,** 1025–1041 (2012).

A7 Membership of the Working Party: A. Hartle (Chair) *et al.* Checking Anaesthetic Equipment 2012: Association of Anaesthetists of Great Britain and Ireland. *Anaesthesia* **67,** 660–668 (2012).

A8 Membership of the Difficult Airway Society Extubation Guidelines Group: M. Popat (Chairman) *et al.* Difficult Airway Society Guidelines for the management of tracheal extubation: Management of tracheal extubation. *Anaesthesia* **67,** 318–340 (2012).

A9 Membership of the Working Party *et al.* Management of proximal femoral fractures 2011: Association of Anaesthetists of Great Britain and Ireland. *Anaesthesia* **67,** 85–98 (2012).

A10 Membership of the Working Party: *et al.* Regional anaesthesia and patients with abnormalities of coagulation: The Association of Anaesthetists of Great Britain & Ireland The Obstetric Anaesthetists' Association Regional Anaesthesia UK. *Anaesthesia* **68,** 966–972 (2013).

A11 Membership of the Working Party: *et al.* Immediate post-anaesthesia recovery 2013: Association of Anaesthetists of Great Britain and Ireland. *Anaesthesia* **68,** 288–297 (2013).

A12 Membership of the Working Party: *et al.* Safety guideline: skin antisepsis for central neuraxial blockade: Association of Anaesthetists of Great Britain and Ireland Obstetric Anaesthetists' Association Regional Anaesthesia UK Association of Paediatric Anaes. *Anaesthesia* **69,** 1279–1286 (2014).

A13 Membership of the Working Party: T. E. Woodcock, Cook, T. M., Gupta, K. J. & Hartle, A. Arterial line blood sampling: preventing hypoglycaemic brain injury 2014: The Association of Anaesthetists of Great Britain and Ireland. *Anaesthesia* **69,** 380–385 (2014).

A14 Membership of the working party: R. Griffiths *et al.* Peri-operative care of the elderly 2014: Association of Anaesthetists of Great Britain and Ireland. *Anaesthesia* **69,** 81–98 (2014).

A15 Mushambi, M. C. *et al.* Obstetric Anaesthetists' Association and Difficult Airway Society guidelines for the management of difficult and failed tracheal intubation in obstetrics. *Anaesthesia* **70,** 1286–1306 (2015).

A16 Members of the Working Party: *et al.* Peri-operative management of the obese surgical patient 2015: Association of Anaesthetists of Great Britain and Ireland Society for Obesity and Bariatric Anaesthesia. *Anaesthesia* **70,** 859–876 (2015).

A17 Membership of the Working Party *et al.* Safety guideline: reducing the risk from cemented hemiarthroplasty for hip fracture 2015: Association of Anaesthetists of Great Britain and Ireland British Orthopaedic Association British Geriatric Society. *Anaesthesia* **70,** 623–626 (2015).

A18 Bodenham Chair, A. *et al.* Association of Anaesthetists of Great Britain and Ireland: Safe vascular access 2016. *Anaesthesia* **71,** 573–585 (2016).

A19 Hartle, A. *et al.* The measurement of adult blood pressure and management of hypertension before elective surgery: Joint Guidelines from the Association of Anaesthetists of Great Britain and Ireland and the British Hypertension Society. *Anaesthesia* **71,** 326–337 (2016).

A20 Klein, A. A. *et al.* AAGBI guidelines: the use of blood components and their alternatives 2016. *Anaesthesia* **71,** 829–842 (2016).

A21 Muñoz, M. *et al.* International consensus statement on the peri-operative management of anaemia and iron deficiency. *Anaesthesia* **72,** 233–247 (2017).

B1. Gan, T. J. *et al.* Society for Ambulatory Anesthesia guidelines for the management of postoperative nausea and vomiting. *Anesth. Analg.* **105,** 1615–1628, table of contents (2007).

B2 Reeves, S. T. *et al.* Guidelines for performing a comprehensive epicardial echocardiography examination: recommendations of the American Society of Echocardiography and the Society of Cardiovascular Anesthesiologists. *Anesth. Analg.* **105,** 22–28 (2007).

B3 Glas, K. E. *et al.* Guidelines for the Performance of a Comprehensive Intraoperative Epiaortic Ultrasonographic Examination: Recommendations of the American Society of Echocardiography and the Society of Cardiovascular Anesthesiologists; Endorsed by the Society of Thoracic Surgeons: *Anesthesia & Analgesia* **106,** 1376–1384 (2008).

B4. Fleisher, L. A. *et al.* ACC/AHA 2006 Guideline Update on Perioperative Cardiovascular Evaluation for Noncardiac Surgery: Focused Update on Perioperative Beta-Blocker Therapy ??? A Report of the American College of Cardiology/American Heart Association Task Force on Practice Guidelines (Writing Committee to Update the 2002 Guidelines on Perioperative Cardiovascular Evaluation for Noncardiac Surgery): *Anesthesia & Analgesia* **104,** 15–26 (2007).

B5 Baranov, D. *et al.* Consensus Statement: First International Workshop on Anesthetics and Alzheimer's Disease: *Anesthesia & Analgesia* **108,** 1627–1630 (2009).

B6 Joshi, G. P. *et al.* Society for Ambulatory Anesthesia Consensus Statement on Perioperative Blood Glucose Management in Diabetic Patients Undergoing Ambulatory Surgery: *Anesthesia & Analgesia* **111,** 1378–1387 (2010).

B7 Hiratzka, L. F. *et al.* 2010 ACCF/AHA/AATS/ACR/ASA/SCA/SCAI/SIR/STS/SVM Guidelines for the diagnosis and management of patients with thoracic aortic disease: Executive summary: A report of the American College of Cardiology Foundation/American Heart Association Task Force on Practice Guidelines, American Association for Thoracic Surgery, American College of Radiology, American Stroke Association, Society of Cardiovascular Anesthesiologists, Society for Cardiovascular Angiography and Interventions, Society of Interventional Radiology, Society of Thoracic Surgeons, and Society for Vascular Medicine. *Anesth. Analg.* **111,** 279–315 (2010).

B8 Troianos, C. A. *et al.* Special articles: guidelines for performing ultrasound guided vascular cannulation: recommendations of the American Society of Echocardiography and the Society Of Cardiovascular Anesthesiologists. *Anesth. Analg.* **114,** 46–72 (2012).

B9 Hillis, L. D. *et al.* 2011 ACCF/AHA Guideline for Coronary Artery Bypass Graft Surgery: Executive Summary. *Anesthesia & Analgesia* **114,** 11–45 (2012).

B10 Lipman, S. *et al.* The Society for Obstetric Anesthesia and Perinatology Consensus Statement on the Management of Cardiac Arrest in Pregnancy: *Anesthesia & Analgesia* **118,** 1003–1016 (2014).

B11 Gan, T. J. *et al.* Consensus Guidelines for the Management of Postoperative Nausea and Vomiting: *Anesthesia & Analgesia* **118,** 85–113 (2014).

B12 Hahn, R. T. *et al.* Guidelines for performing a comprehensive transesophageal echocardiographic examination: recommendations from the American Society of Echocardiography and the Society of Cardiovascular Anesthesiologists. *Anesth. Analg.* **118,** 21–68 (2014).

B13 Politis, G. D. *et al.* Guidelines for Pediatric Perioperative Care During Short-Term Plastic Reconstructive Surgical Projects in Less Developed Nations: *Anesthesia & Analgesia* **112,** 183–190 (2011).

B14 Joshi, G. P., Ankichetty, S. P., Gan, T. J. & Chung, F. Society for Ambulatory Anesthesia Consensus Statement on Preoperative Selection of Adult Patients with Obstructive Sleep Apnea Scheduled for Ambulatory Surgery: *Anesthesia & Analgesia* **115,** 1060–1068 (2012).

B15 Reeves, S. T. *et al.* Basic Perioperative Transesophageal Echocardiography Examination: A Consensus Statement of the American Society of Echocardiography and the Society of Cardiovascular Anesthesiologists. *Journal of the American Society of Echocardiography* **26,** 443–456 (2013).

B16 Chung, F. *et al.* Society of Anesthesia and Sleep Medicine Guidelines on Preoperative Screening and Assessment of Adult Patients With Obstructive Sleep Apnea: *Anesthesia & Analgesia* **123,** 452–473 (2016).

B17 Odegard, K. C. *et al.* SCAI/CCAS/SPA Expert Consensus Statement for Anesthesia and Sedation Practice: Recommendations for Patients Undergoing Diagnostic and Therapeutic Procedures in the Pediatric and Congenital Cardiac Catheterization Laboratory. *Anesth. Analg.* **123,** 1201–1209 (2016).

B18 Shore-Lesserson, L. *et al.* The Society of Thoracic Surgeons, The Society of Cardiovascular Anesthesiologists, and The American Society of ExtraCorporeal Technology: Clinical Practice Guidelines-Anticoagulation During Cardiopulmonary Bypass. *Anesth. Analg.* **126,** 413–424 (2018).

B19 Nasr, V. G. *et al.* Consensus Statement by the Congenital Cardiac Anesthesia Society: Milestones for the Pediatric Cardiac Anesthesia Fellowship. *Anesthesia & Analgesia* **126,** 198–207 (2018).

B20 Memtsoudis, S. G. *et al.* Society of Anesthesia and Sleep Medicine Guideline on Intraoperative Management of Adult Patients With Obstructive Sleep Apnea: *Anesthesia & Analgesia* 1 (2018). doi:10.1213/ANE.0000000000003434

B21 Abola, R. E. *et al.* American Society for Enhanced Recovery and Perioperative Quality Initiative Joint Consensus Statement on Patient-Reported Outcomes in an Enhanced Recovery Pathway. *Anesth. Analg.* **126,** 1874–1882 (2018).

B22 Leffert, L. *et al.* The Society for Obstetric Anesthesia and Perinatology Consensus Statement on the Anesthetic Management of Pregnant and Postpartum Women Receiving Thromboprophylaxis or Higher Dose Anticoagulants: *Anesthesia & Analgesia* **126,** 928–944 (2018).

C1 American Society of Anesthesiologists and Society of Cardiovascular Anesthesiologists Task Force on Transesophageal Echocardiography. Practice guidelines for perioperative transesophageal echocardiography. An updated report by the American Society of Anesthesiologists and the Society of Cardiovascular Anesthesiologists Task Force on Transesophageal Echocardiography. *Anesthesiology* **112,** 1084–1096 (2010).

C2 American Society of Anesthesiologists Task Force on Chronic Pain Management & American Society of Regional Anesthesia and Pain Medicine. Practice guidelines for chronic pain management: an updated report by the American Society of Anesthesiologists Task Force on Chronic Pain Management and the American Society of Regional Anesthesia and Pain Medicine. *Anesthesiology* **112,** 810–833 (2010).

C3 American Society of Anesthesiologists Task Force on Central Venous Access *et al.* Practice guidelines for central venous access: a report by the American Society of Anesthesiologists Task Force on Central Venous Access. *Anesthesiology* **116,** 539–573 (2012).

C4 American Society of Anesthesiologists Task Force on Acute Pain Management. Practice guidelines for acute pain management in the perioperative setting: an updated report

by the American Society of Anesthesiologists Task Force on Acute Pain Management. *Anesthesiology* **116,** 248–273 (2012).

C5 Apfelbaum, J. L. *et al.* Practice guidelines for postanesthetic care: an updated report by the American Society of Anesthesiologists Task Force on Postanesthetic Care. *Anesthesiology* **118,** 291–307 (2013).

C6 Apfelbaum, J. L. *et al.* Practice guidelines for management of the difficult airway: an updated report by the American Society of Anesthesiologists Task Force on Management of the Difficult Airway. *Anesthesiology* **118,** 251–270 (2013).

C7 American Society of Anesthesiologists Task Force on Perioperative Management of patients with obstructive sleep apnea. Practice guidelines for the perioperative management of patients with obstructive sleep apnea: an updated report by the American Society of Anesthesiologists Task Force on Perioperative Management of patients with obstructive sleep apnea. *Anesthesiology* **120,** 268–286 (2014).

C8 Practice Guidelines for Perioperative Blood ManagementAn Updated Report by the American Society of Anesthesiologists Task Force on Perioperative Blood Management*. 35

C9 Practice Guidelines for Obstetric Anesthesia: An Updated Report by the American Society of Anesthesiologists Task Force on Obstetric Anesthesia and the Society for Obstetric Anesthesia and Perinatology. *Anesthesiology* **124,** 270–300 (2016).

C10 Practice Guidelines for the Prevention, Detection, and Management of Respiratory Depression Associated with Neuraxial Opioid AdministrationAn Updated Report by the American Society of Anesthesiologists Task Force on Neuraxial Opioids and the American Society of Regional Anesthesia and Pain Medicine*. 18

C11 Practice Guidelines for Preoperative Fasting and the Use of Pharmacologic Agents to Reduce the Risk of Pulmonary Aspiration: Application to Healthy Patients Undergoing Elective ProceduresAn Updated Report by the American Society of Anesthesiologists Task Force on Preoperative Fasting and the Use of Pharmacologic Agents to Reduce the Risk of Pulmonary Aspiration*. 18

C12 Practice Guidelines for Moderate Procedural Sedation and Analgesia 2018: A Report by the American Society of Anesthesiologists Task Force on Moderate Procedural Sedation and Analgesia, the American Association of Oral and Maxillofacial Surgeons, American College of Radiology, American Dental Association, American Society of Dentist

Anesthesiologists, and Society of Interventional Radiology*. *Anesthesiology* **128,** 437–479 (2018).

D1 Glahn, K. P. E. *et al.* Recognizing and managing a malignant hyperthermia crisis: guidelines from the European Malignant Hyperthermia Group. *British Journal of Anaesthesia* **105,** 417–420 (2010).

D2 Goodnough, L. T. *et al.* Detection, evaluation, and management of preoperative anaemia in the elective orthopaedic surgical patient: NATA guidelines. *British Journal of Anaesthesia* **106,** 13–22 (2011).

D3 Lee, J., Gupta, S., Price, C. & Baranowski, A. P. Low back and radicular pain: a pathway for care developed by the British Pain Society. *British Journal of Anaesthesia* **111,** 112–120 (2013).

D4 Hopkins, P. M. *et al.* European Malignant Hyperthermia Group guidelines for investigation of malignant hyperthermia susceptibility. *British Journal of Anaesthesia* **115,** 531–539 (2015).

D5 Beris, P. *et al.* Perioperative anaemia management: consensus statement on the role of intravenous iron. *British Journal of Anaesthesia* **100,** 599–604 (2008).

D6 Zakrzewska, J. M. Differential diagnosis of facial pain and guidelines for management. *British Journal of Anaesthesia* **111,** 95–104 (2013).

D7 Frerk, C. *et al.* Difficult Airway Society 2015 guidelines for management of unanticipated difficult intubation in adults † †This Article is accompanied by Editorials aev298 and aev404. *British Journal of Anaesthesia* **115,** 827–848 (2015).

D8 Buggy, D. J. *et al.* Consensus statement from the BJA Workshop on Cancer and Anaesthesia. *British Journal of Anaesthesia* **114,** 2–3 (2015).

D9 Higgs, A. *et al.* Guidelines for the management of tracheal intubation in critically ill adults. *British Journal of Anaesthesia* **120,** 323–352 (2018).

D10 Harvey, D. *et al.* Management of perceived devastating brain injury after hospital admission: a consensus statement from stakeholder professional organizations. *British Journal of Anaesthesia* **120,** 138–145 (2018).

D11 Levett, D. Z. H. *et al.* Perioperative cardiopulmonary exercise testing (CPET): consensus clinical guidelines on indications, organization, conduct, and physiological interpretation. *British Journal of Anaesthesia* **120,** 484–500 (2018).

F1 Rochwerg, B. *et al.* CCCS-SSAI WikiRecs Clinical Practice Guideline: vasopressor blood pressure targets in critically ill adults with hypotension. *Can J Anaesth* **64,** 763–765 (2017).

F2 Dobson, G. *et al.* Guidelines to the Practice of Anesthesia - Revised Edition 2018. *Can J Anaesth* **65,** 76–104 (2018).

E1 Cunningham, A. J. *et al.* Guidelines for anaesthesiologist specialist training in pain medicine: SECTION AND BOARD OF ANAESTHESIOLOGY1, European Union of Medical Specialists. *European Journal of Anaesthesiology* **24,** 568–570 (2007).

E2 Knape, J. T. A. *et al.* Guidelines for sedation and/or analgesia by non-anaesthesiology doctors: SECTION and BOARD OF ANAESTHESIOLOGY1, European Union of Medical Specialists. *European Journal of Anaesthesiology* **24,** 563–567 (2007).

E3 Mellin-Olsen, J. *et al.* Guidelines for safety and quality in anaesthesia practice in the European Union: SECTION and BOARD OF ANAESTHESIOLOGY1, European Union of Medical Specialists. *European Journal of Anaesthesiology* **24,** 479–482 (2007).

E4 Alahuhta, S., Mellin-Olsen, J., Blunnie, W. P. & Knape, J. T. A. Charter on continuing medical education/continuing professional development approved by the UEMS Specialist Section and European Board of Anaesthesiology*: *European Journal of Anaesthesiology* **24,** 483–485 (2007).

E5 De Robertis, E., McAdoo, J., Pagni, R. & Knape, J. T. A. Core curriculum in emergency medicine integrated in the specialty of anaesthesiology1: *European Journal of Anaesthesiology* **24,** 987–990 (2007).

E6 Carlsson, C. *et al.* Education and training in Anaesthesia – Revised guidelines by the European Board of Anaesthesiology, Reanimation and Intensive Care: SECTION and BOARD OF ANAESTHESIOLOGY1, European Union of Medical specialists. *European Journal of Anaesthesiology* **25,** 528–530 (2008).

E7 Vimlati, L., Gilsanz, F. & Goldik, Z. Quality and safety guidelines of postanaesthesia care: Working Party on Post Anaesthesia Care (approved by the European Board and Section of Anaesthesiology, Union Européenne des Médecins Spécialistes). *European Journal of Anaesthesiology* **26,** 715–721 (2009).

E8 Dumonceau, J. *et al.* European Society of Gastrointestinal Endoscopy, European Society of Gastroenterology and Endoscopy Nurses and Associates, and the European Society of Anaesthesiology Guideline: Non-anaesthesiologist administration of propofol for GI endoscopy: *European Journal of Anaesthesiology* **27,** 1016–1030 (2010).

E9. Gogarten, W. *et al.* Regional anaesthesia and antithrombotic agents: recommendations of the European Society of Anaesthesiology: *European Journal of Anaesthesiology* **27,** 999–1015 (2010).

E10 Poldermans, D. *et al.* Guidelines for pre-operative cardiac risk assessment and perioperative cardiac management in non-cardiac surgery: the Task Force for Preoperative Cardiac Risk Assessment and Perioperative Cardiac Management in Non-cardiac Surgery of the European Society of Cardiology (ESC) and endorsed by the European Society of Anaesthesiology (ESA). *Eur J Anaesthesiol* **27,** 92–137 (2010).

E11 Smith, I. *et al.* Perioperative fasting in adults and children: guidelines from the European Society of Anaesthesiology. *European Journal of Anaesthesiology* **28,** 556–569 (2011).

E12 De Hert, S. *et al.* Preoperative evaluation of the adult patient undergoing non-cardiac surgery: guidelines from the European Society of Anaesthesiology. *European Journal of Anaesthesiology* **28,** 684–722 (2011).

E13 Sümpelmann, R. *et al.* European consensus statement for intraoperative fluid therapy in children. *Eur J Anaesthesiol* **28,** 637–639 (2011).

E14 Bonhomme, F., Ajzenberg, N., Schved, J.-F., Molliex, S. & Samama, C.-M. Pre-interventional haemostatic assessment: Guidelines from the French Society of Anaesthesia and Intensive Care. *European Journal of Anaesthesiology* **30,** 142–162 (2013).

E15 Kristensen, S. D. *et al.* 2014 ESC/ESA Guidelines on non-cardiac surgery: cardiovascular assessment and management. *European Journal of Anaesthesiology* **31,** 517–573 (2014).

E16. Jammer, I. *et al.* Standards for definitions and use of outcome measures for clinical effectiveness research in perioperative medicine: European Perioperative Clinical Outcome (EPCO) definitions. *European Journal of Anaesthesiology* **32,** 88–105 (2015).

E17 Perel, A. Non-anaesthesiologists should not be allowed to administer propofol for procedural sedation: a Consensus Statement of 21 European National Societies of Anaesthesia. *European Journal of Anaesthesiology* **28,** 580–584 (2011).

E18 Marx, G. *et al.* Intravascular volume therapy in adults: Guidelines from the Association of the Scientific Medical Societies in Germany. *European Journal of Anaesthesiology* **33,** 488–521 (2016).

E19 Aldecoa, C. *et al.* European Society of Anaesthesiology evidence-based and consensus-based guideline on postoperative delirium: *European Journal of Anaesthesiology* **34,** 192–214 (2017).

E20 Kozek-Langenecker, S. A. *et al.* Management of severe perioperative bleeding: guidelines from the European Society of Anaesthesiology. *European Journal of Anaesthesiology* **34,** 332–395 (2017).

E21 Samama, C. M., Afshari, A. & ESA VTE Guidelines Task Force. European guidelines on perioperative venous thromboembolism prophylaxis. *Eur J Anaesthesiol* **35,** 73–76 (2018).

E22 Hinkelbein, J. *et al.* European Society of Anaesthesiology and European Board of Anaesthesiology guidelines for procedural sedation and analgesia in adults. *Eur J Anaesthesiol* **35,** 6–24 (2018).

E23. De Hert, S. *et al.* Pre-operative evaluation of adults undergoing elective noncardiac surgery: Updated guideline from the European Society of Anaesthesiology. *Eur J Anaesthesiol* **35,** 407–465 (2018).

G1 Mashour, G. A., Avitsian, R. & Soriano, S. G. Neuroanesthesiology Fellowship Training: Curricular Guidelines From the Society for Neuroscience in Anesthesiology and Critical Care. *J Neurosurg Anesthesiol* **25,** 7 (2013).

G2 Lele, A. V. *et al.* Perioperative Management of Adult Patients With External Ventricular and Lumbar Drains: Guidelines From the Society for Neuroscience in Anesthesiology and Critical Care. *Journal of Neurosurgical Anesthesiology* **29,** 191–210 (2017).

G3 Talke, P. O., Sharma, D. & Bergese, S. D. Society for Neuroscience in Anesthesiology and Critical Care Expert Consensus Statement: Anesthetic Management of Endovascular Treatment for Acute Ischemic Stroke. *J Neurosurg Anesthesiol* **26,** 14 (2014).

G4 Mashour, G. A., Moore, L. E., Lele, A. V. & Robicsek, S. A. Perioperative Care of Patients at High Risk for Stroke during or after Non-Cardiac, Non-Neurologic Surgery: Consensus Statement from the Society for Neuroscience in Anesthesiology and Critical Care. *J Neurosurg Anesthesiol* **26,** 13 (2014).

H1 Rusticali, B. & Villani, R. Treatment of minor and severe traumatic brain injury. National reference guidelines. *MINERVA ANESTESIOLOGICA* **74,** 34 (2008).

H2 Rocca, G. D. *et al.* II Italian intersociety consensus statement on antithrombotic prophylaxis in orthopedics and traumatology. *MINERVA ANESTESIOLOGICA* **79,** 15 (2013).

H3 Savoia, G. *et al.* SIAARTI – IRC Recommendations for organizing responses to In-Hospital emergencies. *MINERVA ANESTESIOLOGICA* **73,** 21 (2007).

H4 Merli, G. *et al.* Recommendations for airway control and difficult airway management in thoracic anesthesia and lung separation procedures. *MINERVA ANESTESIOLOGICA* **75,** 38 (2009).

H5 Savoia, G. *et al.* Postoperative pain treatment SIAARTI Recommendations 2010 Short version. *MINERVA ANESTESIOLOGICA* **76,** 11 (2010).

H6 Serafini, G. *et al.* Preoperative evaluation in infants and children: recommendations of the Italian Society of Pediatric and Neonatal Anesthesia and Intensive Care (SARNePI). *MINERVA ANESTESIOLOGICA* **80,** 9 (2014).

H7 Calise, F. *et al.* Perioperative antibiotic prophylaxis in adults Outline of the principal recommendations National reference guidelines. *MINERVA ANESTESIOLOGICA* **75,** 5 (2009).

H8 Randelli, F. *et al.* Italian intersociety consensus statement on antithrombotic prophylaxis in hip and knee replacement and in femoral neck fracture surgery. *MINERVA ANESTESIOLOGICA* **79,** 7: 778-92 (2013).

I1 Horlocker, T. T. *et al.* Regional Anesthesia in the Patient Receiving Antithrombotic or Thrombolytic Therapy: American Society of Regional Anesthesia and Pain Medicine Evidence-Based Guidelines (Third Edition). *Regional Anesthesia and Pain Medicine* **35,** 64–101 (2010).

I2 Narouze, S. *et al.* Interventional Spine and Pain Procedures in Patients on Antiplatelet and Anticoagulant Medications: Guidelines From the American Society of Regional Anesthesia and Pain Medicine, the European Society of Regional Anaesthesia and Pain Therapy, the American Academy of Pain Medicine, the International Neuromodulation Society, the North American Neuromodulation Society, and the World Institute of Pain. *Regional Anesthesia and Pain Medicine* **40,** 182–212 (2015).

I3 The Regional Anesthesiology and Acute Pain Medicine Fellowship Directors Group. Guidelines for Fellowship Training in Regional Anesthesiology and Acute Pain Medicine: Third Edition, 2014. *Regional Anesthesia and Pain Medicine* **40,** 213–217 (2015).

I4 Sites, B. D. *et al.* The American Society of Regional Anesthesia and Pain Medicine and the European Society of Regional Anaesthesia and Pain Therapy Joint Committee Recommendations for Education and Training in Ultrasound-Guided Regional Anesthesia: *Regional Anesthesia and Pain Medicine* **34,** 40–46 (2009).

I5 Narouze, S. N. *et al.* The American Society of Regional Anesthesia and Pain Medicine, the European Society of Regional Anaesthesia and Pain Therapy, and the Asian Australasian Federation of Pain Societies Joint Committee Recommendations for Education and Training in Ultrasound-Guided Interventional Pain Procedures: *Regional Anesthesia and Pain Medicine* **37,** 657–664 (2012).

I6 Narouze, S. *et al.* Interventional Spine and Pain Procedures in Patients on Antiplatelet and Anticoagulant Medications (Second Edition): Guidelines From the American Society of Regional Anesthesia and Pain Medicine, the European Society of Regional Anaesthesia and Pain Therapy, the American Academy of Pain Medicine, the International Neuromodulation Society, the North American Neuromodulation Society, and the World Institute of Pain. *Regional Anesthesia and Pain Medicine* **1** (2017).

I7 Horlocker, T. T. *et al.* Regional Anesthesia in the Patient Receiving Antithrombotic or Thrombolytic Therapy: American Society of Regional Anesthesia and Pain Medicine Evidence-Based Guidelines (Fourth Edition). *Regional Anesthesia and Pain Medicine* **43,** 263–309 (2018).
